# Analysis of the impact of health interventions on vaccination coverage for children under two years of age in municipalities of Minas Gerais

**DOI:** 10.1590/1980-549720240028

**Published:** 2024-05-27

**Authors:** Carolina Machado Moreira, Thales Philipe Rodrigues da Silva, Mariana Coelho De Almeida Neves, Marcus Vinicius Gonçalves da Cruz, Elice Eliane Nobre Ribeiro, Silvio Ferreira, Sheila Aparecida Ferreira Lachtim, Fernanda Penido Matozinhos

**Affiliations:** I Universidade Federal de Minas Gerais – Belo Horizonte (MG), Brazil.; II Universidade Federal de São Paulo, Escola Paulista de Enfermagem, Department of Women's Health Nursing – São Paulo (SP), Brazil.; III Universidade Federal de Minas Gerais, School of Nursing, Postgraduate Program in Nursing – Belo Horizonte (MG), Brazil.; IV Faculdade de Ciências Médicas de Minas Gerais – Belo Horizonte (MG), Brazil.; V Fundação João Pinheiro – Belo Horizonte (MG), Brazil.

**Keywords:** Vaccination, Vaccination coverage, Communicable diseases, Counties, Epidemiology, Vacinação, Cobertura vacinal, Doenças transmissíveis, Municípios, Epidemiologia

## Abstract

**Objective:**

To evaluate the impact of the state action-research project on vaccination coverage in children under two years of age in the state of Minas Gerais, according to the size of the municipalities, comparing the years 2021 and 2022.

**Methods:**

This is a study nested within the state action-research project, a before-after community clinical trial carried out in 212 municipalities in the state of Minas Gerais. This study used secondary data on Vaccination Coverage (VC), Homogeneity of Vaccines (HVC) and Abandonment rate of multi-dose vaccines. After classifying municipalities by size and vaccination coverage rates were equitably classified, an analysis of secondary data on 12 immunobiologicals indicated for the age group in question and their abandonment rate of multi-dose vaccines was carried out.

**Results:**

There was an increase in the proportion of municipalities classified as small that reached the vaccination coverage target set by the National Immunization Program (PNI) after the action-research project was carried out. There was an increase in the proportion of small municipalities classified as having a low abandonment rate for the rotavirus vaccine, in the adequate homogeneity of vaccination coverage and in the classification of risk as very low risk and low and medium risk, all with a statistically significant difference.

**Conclusion:**

There was an influence of municipal size on the effectiveness of the actions applied to increase vaccination coverage, explaining that proposing individualized actions for each municipality is essential to improve vaccination coverage.

## RESUMO

**Objetivo:**

Avaliar o impacto do projeto estadual de pesquisa-ação nas coberturas vacinais em crianças menores de dois anos no estado de Minas Gerais, segundo o porte dos municípios, comparando os anos de 2021 e 2022.

**Métodos:**

Estudo aninhado ao projeto estadual de pesquisa-ação, um ensaio clínico comunitário, do tipo antes-depois, realizado em 212 municípios do estado de Minas Gerais. Utilizou-se, neste estudo, dados secundários de Cobertura Vacinal (CV), Homogeneidade das Vacinas (HCV) e Proporção de Abandono (PA). Após a classificação de municípios por porte, e as taxas de cobertura vacinal serem classificadas de maneira equânime, foi realizada uma análise de dados secundários de 12 imunobiológicos indicados para a faixa etária em questão e de suas PAs.

**Resultados:**

Observou-se aumento na proporção de municípios classificados como de pequeno porte que atingiram a meta da cobertura vacinal estabelecida pelo Programa Nacional de Imunização (PNI), após a realização do projeto de pesquisa-ação. Houve aumento na proporção de municípios de pequeno porte classificados como de baixa PA para a vacina Rotavírus, na homogeneidade adequada da CV e na classificação de risco para risco muito baixo e risco baixo e médio, todos com diferença estatisticamente significativa.

**Conclusão:**

Houve influência do porte municipal na efetividade das ações aplicadas para aumento de cobertura vacinal, explicitando que a proposição de ações individualizadas para cada município é imprescindível para melhorar a cobertura vacinal.

## INTRODUCTION

After five decades since the establishment of the National Immunization Program (*Programa Nacional de Imunizações* – PNI), Brazil remains a global benchmark in vaccination efforts, offering 19 immunization agents tailored for various life stages and specific populations. Through comprehensive coordination strategies, PNI has successfully eliminated and eradicated vaccine-preventable diseases nationwide^1,2^, thereby ensuring effective protection for individuals and communities alike, while significantly enhancing the public health landscape.

Moreover, the implementation of PNI has focused on inclusivity and the reduction of regional and social disparities by operating in hard-to-reach areas without discrimination against individuals^1,2^. This underscores the practical application of universality, a cornerstone principle of the Brazilian Unified Health System (*Sistema Único de Saúde* – SUS), which has transformed healthcare into a fundamental right for all^1,2^.

However, it should be noted that the average vaccination coverage in Brazil declined from 97% in 2015 to 75% in 2020 for vaccines such as BCG, Hepatitis B, Poliomyelitis, Human Rotavirus, Pentavalent, Pneumococcal 10 and 13, Meningococcal C, Yellow Fever, and Triple Viral^3^. This decline has led to the concerning resurgence of diseases that were previously eradicated^4-6^.

This decline in vaccination coverage in recent years cannot be attributed to a single reason, as the drop is the result of multiple factors^7-9^. These include a lack of awareness about the seriousness of the situation, insufficient investments in health, structural issues and the circulation of fake news are reasons that possibly work together for the drop in the vaccination rate^8^. It is imperative to implement measures to restore high levels of vaccination coverage in the country^10,11^.

Brazil includes states with vast territorial extensions, such as Minas Gerais, which comprises 853 municipalities, making it the state with the largest number of municipalities in the country. It ranks fourth in terms of territorial area, encompassing extensive urban and rural areas^12^. The decline in vaccination coverage in Minas Gerais has mirrored similar trends observed in other states, particularly concerning the BCG, Polio, and MMR vaccines^12^. Given its vast territorial expanse, Minas Gerais exhibits diverse environmental, cultural, and primarily social contexts. Hence, it is crucial to examine regional and quantitative variations in immunization across each municipality to establish a stratified overview, facilitating the formulation of targeted and uniform interventions^11,12^.

The decline in vaccination coverage among children in Minas Gerais exhibited regional disparities in 2021: the state had 70.5% of municipalities experiencing very low Homogeneity of Vaccination Coverage (HVC). Interestingly, among these municipalities, those with larger populations demonstrated the highest percentages of low coverage^11^. In view of this, health regionalization must act to reduce these disparities and this risk factor for public health in the country and so that there are no setbacks in the achievements of PNI^1,4,13^.

The objective of the study conducted by the Center for Studies and Research in Vaccination of the School of Nursing of Universidade Federal de Minas Gerais (*Observatório em Pesquisa e Estudo em Vacinação da Escola de Enfermagem da Universidade Federal de Minas Gerais* – OPESV-EEUFMG) in collaboration with the State Health Department of Minas Gerais (*Secretaria Estadual de Saúde do Estado de Minas Gerais* – SES-MG) was to evaluate the impact of the ‘Strategies for Increasing Vaccination Coverage in Children Under Two Years of Age in the State of Minas Gerais, Brazil: An Action Research" project on vaccination coverage among children, through execution of workshops and preparation of Action Plans individualized to the reality of each municipality in the state^13,14^. This evaluation aimed to assess changes in vaccination coverage between the years 2021 and 2022, taking into account the size of the municipalities in the state.

## METHODS

The study conducted within the state action research project was designed as a community clinical trial of the before-after type. It was carried out in 212 municipalities belonging to 08 Regional Health Managements and Superintendences (*Gerências e Superintendências Regionais de Saúde* – GRS/SRS) in the state of Minas Gerais, Brazil, designed with the aim of organizing and managing health in the regions of the state^14^. All information was extracted from the National Immunization Program Information System (SIPNI), from 2021 and 2022, available at: <sipni.datasus.gov.br>.

The study encompassed 212 municipalities in Minas Gerais, selected based on their low vaccination coverage and a trend of decreasing coverage observed from 2015 to 2021^12^. Workshops were conducted in collaboration with professionals responsible for vaccination and management in each municipality, aiming to develop and monitor action plans tailored to the specific context of each location. The health regions covered and the number of municipalities in each of them were: Regional Health Management of São João del Rey, made up of 20 municipalities; Alfenas Regional Health Superintendence, made up of 24 municipalities; Passos Regional Health Superintendence, made up of 27 municipalities; Leopoldina Regional Health Management, made up of 15 municipalities; Barbacena Regional Health Superintendence, made up of 31 municipalities; Ituiutaba Regional Health Management, made up of 9 municipalities; Regional Health Superintendency of Coronel Fabriciano, made up of 35 municipalities and Regional Health Superintendence of Governador Valadares, made up of 51 municipalities^14^.

The variables listed in Chart 1 were employed in this study. The 212 municipalities under evaluation were categorized according to the classification by Braz et al.^10^, which relies on criteria outlined in the indicators booklet of the Health Surveillance Actions Qualification Program (*Programa de Qualificação das Ações de Vigilância em Saúde* – PQAVS). Namely:

- Small municipality: One with a population of up to 20,000 inhabitants;
- Medium-sized municipality: One with a population between 20,001 and 100,000 inhabitants; and
- Large municipality: One with more than 100,000 inhabitants.

**Chart 1:** Summary of the parameters used to calculate the immunization indicators in this study.

Indicator	Parameters
Vaccination Coverage Index (VCI)	Very low: <50%
	Low: ≥ 50% and lower than target
	Adequate: ≥ target
Homogeneity of Vaccination Coverage (HVC)	Adequate: ≥75% to ≤100%
	Low: ≥50% to <75%
	Very low: <50%
Abandonment rate of multi-dose vaccines	Low: <5%
	Medium: ≥5% to <10%
	High: ≥10%
Population size	Small size: Population ≤ 20,000 inhabitants
	Medium size: Population between 20,001 and 100,000 inhabitants
	Large size: Population ≥ 100,001 inhabitants
Risk Classification of Transmission of Vaccine-Preventable Diseases	Very low: municipalities with HVC =100%
	Low: municipalities with HVC ≥75% and <100%, with adequate VCI for Polio, MMR and Pentavalent vaccines
	Medium: municipality with HVC ≥75% and <100% and VCI below the target for one or more of the Polio, MMR or Pentavalent vaccines
	High: municipalities with HVC <75%, regardless of vaccination coverage
	Very high: municipality with HVC <75%, high TA (≥10%) for any of the vaccines evaluated and with a large population size and, also, municipalities without vaccination records for any vaccine, regardless of population size

In this study, secondary data on Vaccination Coverage (VC), Homogeneity of Vaccination Coverage (HVC), and Abandonment rate of multi-dose vaccines for 12 immunobiologicals recommended for children under two years of age were utilized. The data spanned from January to December 2021 (pre-intervention period, prior to the state action research project) and January to December 2022 (post-intervention period).

The 12 analyzed immunobiologicals recommended for children under two years of age were: BCG, Hepatitis B (for children under one month of age), oral vaccine against rotavirus (second dose of Rotavirus vaccine in SUS and the second dose of Pentavalent Rotavirus in the private network), Meningococcal C (second dose of Meningococcal C and second dose of Meningococcal ACWY), Pneumococcal 10 and 13 (two doses), Pentavalent (third dose of the Pentavalent vaccine and third dose of the Hexavalent vaccine in the private network), Polio vaccine (third dose of Pentavalent and Hexavalent VIP/VOP in the private network), Triple Viral (two doses: second dose of Quadruple Viral, second dose, and single dose of Tetraviral), Yellow Fever (double dose, initial and first dose), Hepatitis A (considered one dose), and Chickenpox (1 dose of Chickenpox and 1 dose of Tetraviral).

For VC, the classification of vaccination coverage established by PNI was adopted, according to which coverage must be greater than or equal to 90% for the BCG and oral Human Rotavirus vaccines and greater than or equal to 95% for the other immunizers. Vaccines are considered to have coverage very low if there is a VC between 0 and 50%; low if it is in the range of 50% to any value below the target; and adequate if it is greater than or equal to the target^10^.

HVC, an estimate of the proportion of municipalities with adequate coverage, agreed upon by the Organizational Contract for Public Health Action (*Contrato Organizativo de Ação Pública da Saúde* – COAP)^10^, is considered adequate when the municipality presents: from 75 to 100% of adequate coverage of at least ten vaccines; low when it presents between 50 and less than 75% adequate coverage of at least ten vaccines; and very low when the percentage reaches less than 50%^10^.

The abandonment rate of multi-dose vaccines, which contain multiple doses (such as the following vaccines: Pneumococcal 10 and 13, oral Human Rotavirus vaccine, and VIP/VOP Hexa and Pentavalent against Poliomyelitis), was also explored to understand the scenario of 2021 and 2022 and changes in relation to the abandonment of vaccination in municipalities through classification. It was considered low AP when less than 5% of people (<5%) stopped taking other doses; average when dropout varied from 5% to 10% (≥5 to <10%); and high when it reached a proportion greater than 10%.

Finally, the risk of the return of these diseases in these same municipalities was observed using the classification made by Braz et al.^10^, as follows:

- Very low risk: municipalities with 100% homogeneity;
- Low risk: when there is adequate homogeneity (≥75 and <100%) of Polio, MMR and Pentavalent vaccines;
- Medium risk: ≥75 and 100% of homogeneity and adequate vaccination coverage for one of the three indicated vaccines;
- High risk: homogeneity <75%, regardless of indicator vaccines;
- Very high risk: homogeneity <75% and proportion greater than 10% of abandonment rate of multi-dose vaccines evaluated in large municipalities, in places that do not have vaccination records, regardless of the number of inhabitants

Due to the low number of municipalities that present medium and very high risk, according to the proposed classification, in this study the categories were combined into: "low and medium risk" and "high and very high risk."

The analyses were conducted using the Statistical Software for Professionals (Stata), version 16.0. The aim was to understand the proportion of municipalities with low vaccination coverage for each of the 12 vaccine-preventable diseases analyzed, the proportion of homogeneity, and the percentage risk of vaccine-preventable diseases returning, depending on the size of municipalities. Additionally, the McNemar test was applied to verify the proportions of changes and their relevance, with a significance level set at 5%.

## RESULTS

Following the implementation of the action research project, there was a notable increase in the proportion of municipalities classified as small that met the vaccination coverage targets established by the PNI. Significant increases (p<0.05) were observed for several immunobiologicals: BCG (25.71% to 42.37%), Rotavirus (45.76% to 57.63%), Meningococcus (35.59% to 52.54%), Pneumococcal C (38.98% to 53.11%), Pentavalent (37.85% to 52.54%), Poliomyelitis (38.98% to 53.11%), MMR D2 (22.86% to 35.80%), and Chickenpox (40.11% to 59.89%), as detailed in Table 1.

**Table 1:** Percentage of municipalities according to vaccination coverage classification, Minas Gerais, 2021 and 2022.

		2021	2022	p-value
		% of municipalities that reached the target	% of municipalities that reached the target	
Small-sized municipalities (n=177)				
	BCG	25.71	42.37	**<0.001**
	Hepatitis B	17.51	23.16	0.131
	Rotavirus	45.76	57.63	**0.021**
	Meningococcal C	35.59	52.54	**0.001**
	Pneumococcal C	38.98	53.11	**0.008**
	Penta (DTP/Hib/HB)	37.85	52.54	**0.006**
	Polio	38.98	53.11	**0.008**
	MMR D1	46.33	55.93	0.077
	Yellow Fever	33.33	34.46	0.816
	MMR D2	22.86	35.80	**0.002**
	Hepatitis A	42.94	50.85	0.126
	Varicella	40.11	59.89	**<0.001**
Medium-sized municipalities (n=28)				
	BCG	7.14	39.29	**0.002**
	Hepatitis B	3.57	17.86	**0.045**
	Rotavirus	17.86	28.57	0.256
	Meningococcal C	7.14	14.29	0.317
	Pneumococcal C	10.71	25.00	0.102
	Penta (DTP/Hib/HB)	2.14	14.29	0.317
	Polio	7.14	14.29	0.317
	MMR D1	14.29	17.86	0.654
	Yellow Fever	7.14	7.14	1.000
	MMR D2	3.57	10.71	0.317
	Hepatitis A	3.57	14.29	0.179
	Varicella	3.57	21.43	0.058
Large-sized municipalities (n=7)				
	BCG	14.29	57.14	0.083
	Hepatitis B	14.29	0	0.317
	Rotavirus	0	14.29	0.317
	Meningococcal C	0	28.57	0.157
	Pneumococcal C	0	28.57	0.157
	Penta (DTP/Hib/HB)	0	28.57	0.157
	Polio	0	28.57	0.157
	MMR D1	0	14.29	0.317
	Yellow Fever	0	0	–
	MMR D2	0	0	–
	Hepatitis A	0	14.19	0.317
	Varicella	0	0	–

Note: 212 municipalities; target ≥ 90% for the BCG and Human Rotavirus vaccine and ≥ 95% for other immunobiologicals. 90% for the BCG and Oral Human Rotavirus vaccine and greater than or equal to 95% for other immunizers. Bold indicates statistically significant p-values.

For medium-sized municipalities, there was an increase in the number of municipalities reaching the target vaccination coverage after the survey, notably for BCG and Hepatitis B (first dose), with statistically significant differences observed. Conversely, among municipalities classified as large, there was no statistically significant increase in achieving the vaccination coverage target among children under two years of age (Table 1).

Tables 2, 3, and 4 illustrate the proportions of abandonment rate of multi-dose vaccines, HVC, and risk classification based on the population size of the municipalities. Significantly, among small municipalities, there was an increase in the proportion of those classified as having low abandonment rate of multi-dose vaccines for the Rotavirus vaccine (63.28% to 80.79%) after the implementation of the action research project. Moreover, there was an increase in the number of municipalities categorized with adequate homogeneity in vaccination coverage. Additionally, there was an increase in the number of municipalities classified as very low risk, low risk, and medium risk, coupled with a decrease in those classified as high risk and very high risk, all demonstrating statistically significant differences (Table 2).

**Table 2:** Proportion of abandonment, homogeneity of vaccines and risk classification for the transmission of vaccine-preventable diseases, municipalities with a small population, Minas Gerais, 2021 and 2022.

Small-sized municipalities		2021	2022	p-value
		% of municipalities	% of municipalities	
Abandonment rate of multi-dose vaccines				
Human Rotavirus Oral Vaccine				
	Low (<5%)	63.28	80.79	**<0.001**
	Medium (≥5 to ≤10%)	17.51	11.30	
	High (>10%)	19.21	7.91	
Pneumococcal 10 and 13				
	Low (<5%)	64.97	71.75	0.174
	Medium (≥5 to ≤10%)	15.25	14.69	
	High (>10%)	19.77	13.56	
Pentavalent and Hexavalent				
	Low (<5%)	62.71	60.45	0.679
	Medium (≥5 to ≤10%)	11.86	18.08	
	High (>10%)	25.42	21.47	
IPV/OPV, Hexavalent, and Pentavalent				
	Low (<5%)	58.76	60.45	0.750
	Medium (≥5 to ≤ 10%)	13.56	16.38	
	High (>10%)	27.68	23.16	
Homogeneity of vaccination coverage				
	Adequate for COAP (≥75 to <100%)	23.16	36.16	**0.005**
	Low (≥50 to < 75%)	18.64	18.08	
	Very low (≥0 to <50%)	58.19	45.76	
Risk classification				
	Very low risk	10.73	17.51	**0.039**
	Low and medium risk	12.43	18.64	
	High and very high risk	76.84	63.84	

Bold indicates statistically significant values.

**Table 3:** Proportion of abandonment, homogeneity of vaccines and risk classification for the transmission of vaccine-preventable diseases, municipalities with a medium population size, Minas Gerais, 2021 and 2022.

Medium-sized municipalities		2021	2022	p-value
		% of municipalities	% of municipalities	
Abandonment rate of multi-dose vaccines				
Human Rotavirus Oral Vaccine				
	Low (<5%)	85.71	92.86	0.414
	Medium (≥5 to ≤10%)	14.29	7.14	
	High (>10%)	–		
Pneumococcal 10 and 13				
	Low (<5%)	82.14	92.86	0.256
	Medium (≥5 to ≤10%)	17.86	7.14	
	High (>10%)	–		
Pentavalent and Hexavalent				
	Low (<5%)	75.00	64.29	0.365
	Medium (≥5 to ≤10%)	25.00	21.43	
	High (>10%)		14.29	
IPV/OPV, Hexavalent, and Pentavalent				
	Low (<5%)	71.43	64.29	0.527
	Medium (≥5 to ≤ 10%)	25.00	25.00	
	High (>10%)	3.57	10.71	
Homogeneity of vaccination coverage				
	Adequate for COAP (≥75 to <100%)	–	10.71	0.083
	Low (≥50 to < 75%)	10.71	3.57	
	Very low (≥0 to <50%)	89.29	85.71	
Risk classification				
	Very low risk	–	–	-
	Low and medium risk	–	10.71	
	High and very high risk	100	89.29	

**Table 4:** Proportion of abandonment, homogeneity of vaccines and risk classification for the transmission of vaccine-preventable diseases, municipalities with a large population, Minas Gerais, 2021 and 2022.

Large-sized municipalities		2021	2022	p-value
		% of municipalities	% of municipalities	
Abandonment rate of multi-dose vaccines				
Human Rotavirus Oral Vaccine				
	Low (<5%)	85.71	85.71	-
	Medium (≥5 to ≤10%)	14.29	14.29	
	High (>10%)	–		
Pneumococcal 10 and 13				
	Low (<5%)	85.71	71.43	0.563
	Medium (≥5 to ≤10%)	14.29	28.57	
	High (>10%)	–		
Pentavalent and Hexavalent				
	Low (<5%)	57.14	57.14	1.000
	Medium (≥5 to ≤10%)	14.29	14.29	
	High (>10%)	28.57	28.57	
IPV/OPV, Hexavalent, and Pentavalent				
	Low (<5%)	57.14	57.14	1.000
	Medium (≥5 to ≤ 10%)	14.29	14.29	
	High (>10%)	28.57	28.57	
Homogeneity of vaccination coverage				
	Adequate for COAP (≥75 to <100%)	–		-
	Low (≥50 to < 75%)	–	14.29	
	Very low (≥0 to <50%)	100	85.71	
Risk classification				
	Very low risk	–	–	-
	Low and medium risk	–	–	
	High and very high risk	100	100	

No statistically significant difference was observed between municipalities classified as medium and large for any of the indicators evaluated in this study (Tables 3 and 4).

## DISCUSSION

This study reaffirmed the importance of the activities promoted by the action research project, especially in small municipalities across all regions, while also noting the maintenance of the risk status in some municipalities. Rare exceptions showed a worsening in vaccination coverage. In light of these findings, it was pertinent to analyze the aspects that contributed to this outcome, with the aim of applying them in municipalities that have not yet reached the appropriate vaccination levels.

The extensive connection between primary health care and small municipalities may contribute to the effectiveness of actions in these cities^15^, given that approximately 177 of the municipalities studied have fewer than 20,000 inhabitants. According to the National Primary Care Policy (*Política Nacional de Atenção Básica* – PNAB)^16^, in areas characterized by significant territorial dispersion, risk, and social vulnerability, it is recommended to achieve 100% coverage of the population with a maximum of 750 people per Community Health Worker (CHW).

Moreover, CHWs play an extremely crucial role in transforming this scenario, engaging in health education through both individual and collective activities, and serving as a vital link between the professional team and the community^16^. With their intimate knowledge of the territory and its residents, CHWs can tailor interventions more effectively^17^. Training these CHWs to actively seek out and engage with individuals who are hesitant about vaccination, thus encouraging vaccination uptake, is essential. This is because CHWs are already familiar with local needs and have earned the trust of community members^18^. Therefore, in smaller municipalities, communication becomes more manageable as distances are shorter, and the number of individuals to be reached and informed is lower.

Small municipalities possess highly advantageous characteristics in line with the operational principles of SUS, particularly regionalization, which entails the decentralization of health services and actions tailored to the specific needs of each area, reflecting its historical and cultural context^19^. Consequently, addressing the healthcare needs of small towns becomes more achievable as it allows local authorities to have a deeper understanding of their community's dynamics. This aligns with the strategies employed in the action research, where each region tailored interventions based on identified weaknesses and strengths as highlighted by municipal managers. These interventions included securing funding, extending vaccination service hours, conducting outreach campaigns, and organizing professional development activities. Hence, the physical proximity inherent in small municipalities likely played a pivotal role in the observed improvements within a single year^20^.

The efficacy of interventions in medium and large municipalities also had a notable impact on vaccination coverage across Minas Gerais. Despite these advancements, sustaining progress in certain areas remains a challenge for further enhancing vaccination coverage among children under two years old and addressing apprehensions regarding the resurgence of vaccine-preventable diseases.

Limitations were observed concerning medium and large municipalities, as the improvements achieved were significant but still inadequate to decrease the percentages, unlike what was observed in small municipalities. This underscores weaknesses in the plans devised by regional authorities for these municipalities or in their implementation. Therefore, further investigations are necessary to gain a better understanding of the situation in these locations, as positive results were also observed in some municipalities within each of these regions.

In contrast to small municipalities, medium and large municipalities face challenges related to high population density. The population volume appears to be a complicating element in that it prevents the link between population and service, since, due to there being a large flow, health units tend to be unable to manage the quantity and possible issues that are obstacles in relation to vaccination, resulting in a failure in the responsibility of primary care in this aspect^21^. This burden on health professionals and CHWs affects the implementation of crucial actions like active search, which is more feasible in municipalities with smaller populations. Therefore, the failure to address these challenges and communicate them effectively to relevant authorities perpetuates the decline in vaccination coverage. It is essential to tailor interventions to the specific needs of each location to address territorial demands effectively and achieve positive outcomes across the state.

As vaccination coverage increased, municipalities also improved their risk status, accompanied by a decrease in the proportion of abandonment of multi-dose immunobiologicals. With more individuals vaccinated, the likelihood of diseases resurfacing diminishes^21^, and the awareness fostered — which contributed to the uptick in vaccination rates — may be correlated with the decline in abandonment of multi-dose vaccines.

It is important to acknowledge that this study has certain limitations. One such limitation is the reliance on data provided by SIPNI, which may be subject to underestimation due to potential shortcomings in the system's record-keeping.

Nevertheless, the methodological rigor employed in this study underscores the significance of a tailored approach to each municipality, with the goal of amplifying the positive outcomes observed in vaccination coverage improvement across Minas Gerais. Additionally, it is imperative for regional authorities to draw insights from the successful management strategies implemented in small municipalities and develop effective plans tailored to the unique characteristics of each locality, particularly those with persistently low vaccination coverage rates.
